# Light-chain cardiac amyloidosis for the non-expert: pearls and pitfalls

**DOI:** 10.1007/s11739-023-03335-3

**Published:** 2023-06-20

**Authors:** Laura De Michieli, Giulio Sinigiani, Monica De Gaspari, Antonio Branca, Stefania Rizzo, Cristina Basso, Livio Trentin, Sabino Iliceto, Martina Perazzolo Marra, Alberto Cipriani, Tamara Berno

**Affiliations:** 1 Department of Cardiac, Thoracic and Vascular Sciences and Public Health, University of Padova, Via Giustiniani, 2, 35128 Padua, Italy; 2 Cardiovascular Pathology Unit, University Hospital of Padua, Padua, Italy; 3 Hematology and Clinical Immunology Branch, Department of Medicine, University of Padova, Padua, Italy; 4 Cardiology Unit, University Hospital of Padua, Padua, Italy

**Keywords:** Cardiac amyloidosis, AL amyloidosis, Diagnostic pitfalls

## Abstract

Cardiac amyloidosis (CA) is an uncommon, progressive, and fatal disease; the two main forms that can affect the heart are transthyretin CA and light chain CA (AL-CA). AL-CA is a medical urgency for which a diagnostic delay can be catastrophic for patients’ outcome. In this manuscript, we focus on the pearls and pitfalls that are relevant to achieve a correct diagnosis and to avoid diagnostic and therapeutical delays. Through the aid of three unfortunate clinical cases, some fundamental diagnostic aspects are addressed, including the following: first, a negative bone scintigraphy does not exclude CA, with patients with AL-CA frequently showing no or mild cardiac uptake, and its execution should not delay hematological tests; second, fat pad biopsy does not have a 100% sensitivity for AL amyloidosis and, if negative, further investigations should be performed, particularly if the pre-test probability is high. Third, Congo Red staining is not sufficient to reach a definitive diagnosis and amyloid fibrils typing with mass spectrometry, immunohistochemistry, or immunoelectron microscopy is crucial. To achieve a timely and correct diagnosis, all the necessary investigations must be performed, always considering the yield and diagnostic accuracy of each examination.

## Introduction

Systemic amyloidosis is an uncommon and progressive disease that, in the recent years, has been the subject of a medical revolution in terms of diagnostic and therapeutic advancements. This has been mainly related to the greater availability of non-invasive diagnostic strategies [1, 2] and novel effective therapies for the two most common forms that can affect the heart, such as the immunoglobulin light chains amyloidosis (AL) and transthyretin amyloidosis (ATTR) [3–5].

ATTR (both variant, ATTRv, and wild type, ATTRwt) and AL amyloidosis constitute substantially different conditions. ATTR is caused by misfolding of transthyretin, either due to destabilizing genetic variants in ATTRv or to complex and not completely elucidated mechanisms, including ageing, in ATTRwt [6]. Transthyretin instability, with dissociation into dimers and monomers with subsequent misfolding and amyloid fibers formation, appears to be related to oxidative modifications, age-related failure of cellular homeostatic mechanisms, metal cations and genetic mutations, with a proteolytic pathway also potentially involved [6]. Cardiac involvement is frequent in ATTRv (depending on the causative mutation) and ubiquitous in ATTRwt, and its severity is associated with disease outcome. The diagnosis of ATTR cardiac amyloidosis (CA) can be based either on histological demonstration of ATTR fibrils deposition or on a non-invasive approach in selected cases when AL amyloidosis has been excluded [1].

In AL amyloidosis, a plasma cell clone, or rarely a lymphoplasmacytic or marginal zone lymphoma, produces abnormal and toxic light chains that aggregate to form insoluble fibrils, with deposition in tissues and organ dysfunction [7, 8]. All organs, except the brain, can be involved, with heart and kidneys most frequently affected; this multiorgan involvement accounts for the variable clinical presentation, frequently with nonspecific signs and symptoms [9]. For the diagnosis, monoclonal protein assessment should be performed including serum free light chains (FLCs) measurement and serum and urine protein electrophoresis with immunofixation [10]. Both lambda and kappa light chains can be involved and the difference between involved and uninvolved free light chain (dFLC) has prognostic significance [11].

Histological demonstration of amyloid deposits, with identification of AL type with mass spectrometry, immunohistochemistry or immunoelectron microscopy, is required to achieve a final diagnosis of AL amyloidosis [1]. Cardiac involvement, the severity of which is defined by cardiac troponin (cTn) and natriuretic peptides values [12], is a major determinant of prognosis with median survival < 1 year in AL patients with advanced cardiac disease [12, 13]. The diagnosis of AL cardiac amyloidosis (AL-CA) can be determined based on laboratory, echocardiographic and/or cardiac magnetic resonance (CMR) criteria together with cardiac or extracardiac histological demonstration of AL amyloid deposits [1, 13].

AL amyloidosis can develop in patients with multiple myeloma in 10–15% of cases or in patients with monoclonal gammopathy of undetermined significance (MGUS) in 9% of cases [13]. Therefore, it is recommended to screen for pre-symptomatic amyloid organ involvement in these patients with measurements of brain natriuretic peptide (BNP) or N-terminal pro-brain natriuretic peptide (NT-proBNP), albuminuria and alkaline phosphatase [3]. In the suspicion of initial cardiac involvement, CMR is a useful tool for early diagnosis [14].

Early recognition of AL amyloidosis remains a critical issue. As many as one third of patients with amyloidosis may visit five or more physicians before diagnosis [15], and treatment efficacy is strictly related to patients’ stage at diagnosis [3]. Failure of a timely recognition of AL amyloidosis is catastrophic for patients’ outcome. We report here three clinical cases to point out some crucial diagnostic errors and pitfalls that can contribute to delay in diagnosis and reduced survival.

## Case reports

### A negative bone scintigraphy does not exclude cardiac amyloidosis

**Fig. 1:**
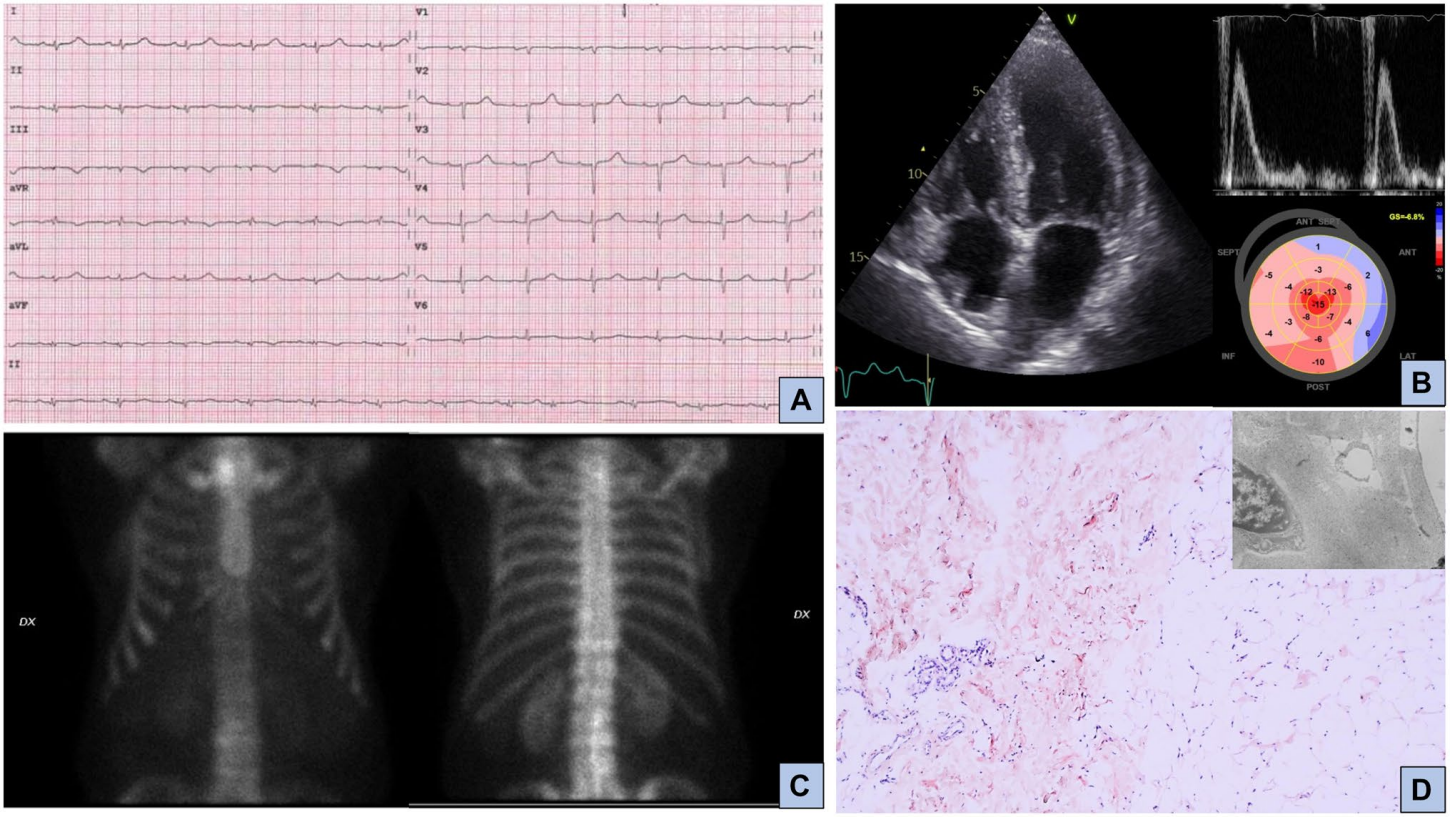
AL amyloidosis with cardiac involvement and negative 99^m^technetium-labelled bone scintigraphy. Panel **A**: 12-lead electrocardiogram showing sinus rhythm, low peripheral QRS voltages, and anterior pseudo necrosis; panel **B**: echocardiographic findings; left panel, apical four chamber view with biventricular hypertrophy and thickened mitral and tricuspid valves leaflets; right upper panel, restrictive left ventricular filling pattern; right lower panel, reduced global longitudinal strain with apical sparing pattern; panel **C**: bone scintigraphy with 99mTc-Hydroxymethylene diphosphonate (HMDP) showing no cardiac uptake; panel **D**: fat pad biopsy positive for Congo red staining and, in the smaller panel, immunoreactivity for lambda light chain at immunogold electron microscopy

Sixty-nine-year-old woman in good health till hospital admission for pulmonary edema and heart failure during a hypertension emergency. Left ventricular hypertrophy was noted at echocardiography, together with reduced global longitudinal strain with relative apical sparing [16], and CMR findings were suggestive for CA. She was discharged and underwent bone scintigraphy 1 month later, which was negative for cardiac uptake (Fig. 1). No further diagnostic testing for amyloidosis was (erroneously) performed. A few months later, she was admitted for acute stroke; at blood tests, there was evidence of IgG lambda monoclonal component, serum FLC lambda were elevated (408 mg/L, dFLC 386 mg/L) with lambda Bence Jones proteinuria. After bone marrow biopsy, a diagnosis of multiple myeloma was achieved. Moreover, she underwent fat pad biopsy that resulted positive for Congo red staining; subsequent typing with immunoelectron microscopy demonstrated lambda-type AL amyloid. Cardiac biomarkers were severely elevated (high-sensitivity cTn T, hs-cTnT, 115 ng/L, NT-proBNP 12540 ng/L, revised Mayo Clinic stage IV [11]). Specific treatment was started, but the patient died after 4 months for refractory heart failure.

### Fat pad biopsy does not have a 100% sensitivity for systemic amyloidosis, and life-saving therapies cannot be precluded just based on this finding

**Fig. 2:**
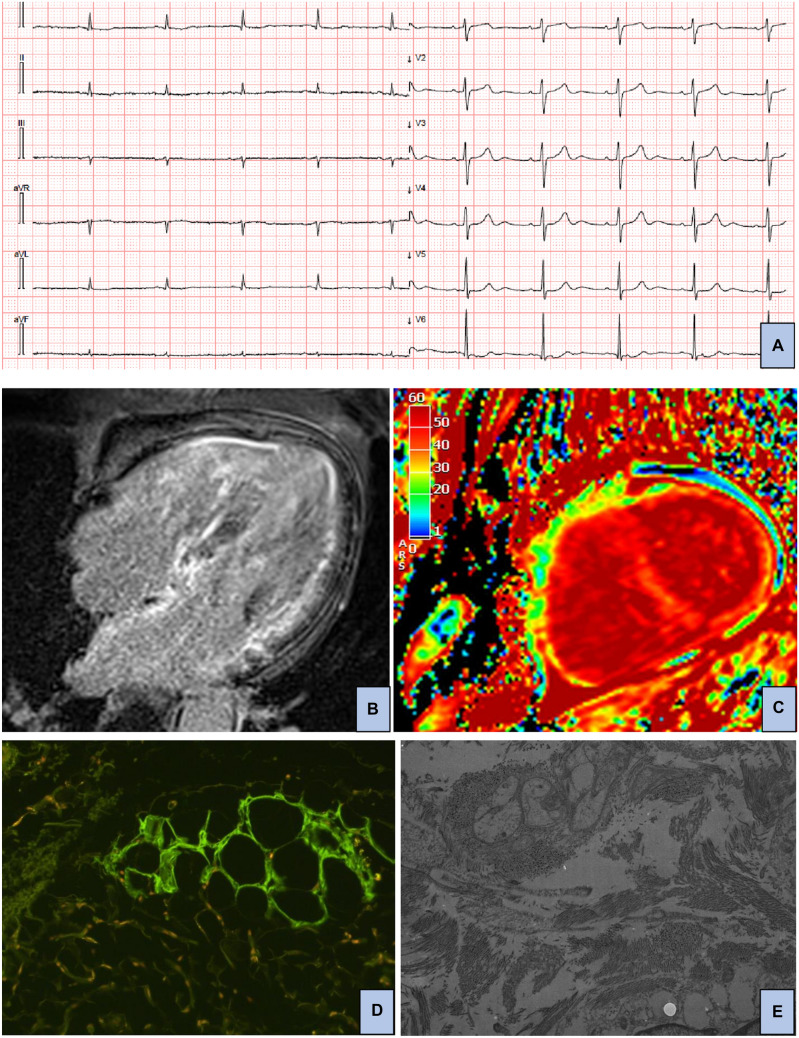
AL amyloidosis with cardiac involvement: electrocardiographic, CMR and histological findings. Panel **A**: 12-lead electrocardiogram with low peripheral QRS voltages; panel **B**: CMR four chamber view image showing biventricular and atrial diffuse late gadolinium enhancement; panel **C**: CMR midventricular short-axis extracellular volume mapping image demonstrating increased values (48%), indicating relevant myocardial infiltration; panel **D**: abdominal fat pad biopsy showing fluorescence at thioflavin staining focally; panel **E**: electron micrograph showing immunoreactivity for lambda light chain of amyloid fibrils, as demonstrated by a post-embedding immunogold method in keeping with light-chain amyloidosis (magnification × 20.0 K)

Man of 49 years in good health till he developed lower limbs paresthesia and weight loss. He visited various physicians without a definitive diagnosis for 10 months. He was subsequently admitted for suspected acute coronary syndrome, which was ruled out with a coronary angiography. Echocardiography showed left ventricular hypertrophy and CMR revealed typical CA findings, such as abnormal gadolinium kinetics, myocardial late gadolinium enhancement “zebra” pattern and increased extracellular volume [17] (Fig. 2). Fat pad biopsy was performed, and Congo Red staining was negative for amyloid deposits. Therefore, amyloidosis diagnosis was (erroneously) excluded, and the patient was discharged. Three months later, he was admitted for heart failure. Serum FLC lambda were elevated (sFLC lambda 780 mg/L, dFLC 764, k/λ ratio 0.02), so were the cardiac biomarkers (hs-cTnI 1336 ng/L, NT-proBNP 35000 ng/L). With this finding, fat pat biopsy was repeated, and it resulted positive for Congo red staining (subsequently typed as lambda-type AL amyloid); bone marrow biopsy was also performed with documentation of 9% plasma cells. Based on cardiac imaging, clinical presentation, and overall laboratory testing (with 24-h urine protein > 0.5 g/day), a diagnosis of AL amyloidosis with cardiac, renal, and neurological involvement was achieved and specific therapy was initiated, though ineffective in preventing disease progression and death of the patient a few weeks later.

### Congo red staining is not enough to start aggressive and potentially hazardous chemotherapy

**Fig. 3:**
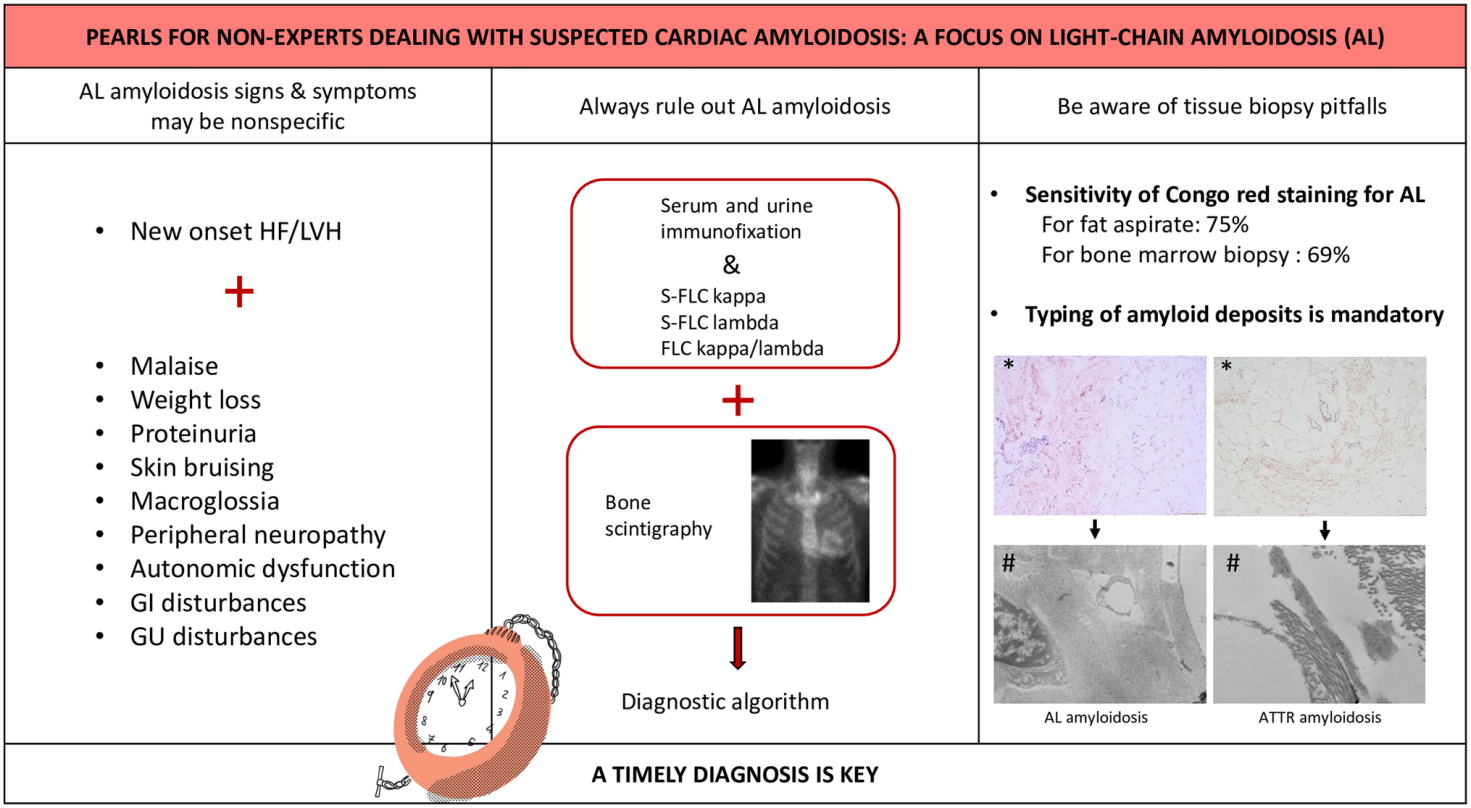
Pearls for non-experts dealing with suspected cardiac amyloidosis: a focus on light-chain amyloidosis (AL). *AL* light chain amyloidosis, *HF* heart failure, *LVH* left ventricular hypertrophy, *GI* gastrointestinal, *GU* genitourinary, *s-FLC* serum free light chain, *ATTR* transthyretin amyloidosis. *Congo red staining; #immunogold electron microscopy. AL amyloidosis fat pad biopsy as in Fig. 1

Seventy-one-year-old man, with previous history of bilateral carpal tunnel syndrome, biceps tendon rupture, IgA kappa MGUS and left ventricular hypertrophy on echocardiography for 3 years. He was hospitalized for heart failure; a CMR was suggestive for infiltrative cardiomyopathy and a subsequent bone scintigraphy showed mild cardiac uptake (Perugini grade 1 [18]). Following laboratory investigations, fat pad biopsy was performed, and amyloid was detected on Congo red staining. Bone marrow aspirate showed 11% of plasma cells infiltration and AL-CA was (erroneously) diagnosed; the patient was sent to the closest CA center for specific chemotherapy. However, fat pad biopsy specimen was re-analyzed with immunogold electron microscopy and transthyretin was identified as the amyloid fibrils precursor (Fig. 3). Specific treatment with transthyretin stabilizer was initiated accordingly**.**

## Discussion

Recent international Consensus Documents [1, 5] and Guidelines [19, 20] have underlined the possibility of non-invasive diagnostic algorithms for ATTR-CA in selected patients. However, every physician dealing with suspected CA should keep in mind that the time-sensitive matter in the diagnostic algorithm is the exclusion of AL amyloidosis, since diagnostic delay of this condition can result in treatment inefficacy and early patients’ death. Both the European and the American Consensus Statements [1, 5] underscore the need for laboratory hematological investigations together with 99^m^technetium-labelled bone scintigraphy, and tissue biopsy if necessary, to achieve a definitive diagnosis. Furthermore, in the American Heart Association Scientific Statement [5] and in the more recent American Heart Failure Guidelines [19], the diagnostic algorithm for suspected CA depicts as the first step the search for a monoclonal component. Only after, or together with, the exclusion of a monoclonal component (with sFLC measurement and serum and urine protein electrophoresis with immunofixation), bone scintigraphy or CMR can be performed and interpreted to achieve the final diagnosis. This is to further stress that, while both laboratory investigations and bone scintigraphy might be necessary for a definitive diagnosis, there should not be any delay or indecision in ruling out AL amyloidosis. Through the presentation of three clinical cases, we report here important errors in the diagnostic process of CA, to raise awareness of the diagnostic pitfalls that can be encountered when dealing with these patients. The three clinical cases were not particularly challenging from a diagnostic standpoint, but some crucial steps in the diagnostic algorithm were misinterpreted or not completely executed, leading to diagnostic delay or misdiagnosis.

As underlined in the first tragic clinical case report, patients with AL-CA most frequently show no or mild (grade 1) uptake at bone scintigraphy [21] (Fig. 1). In the presence of echocardiographic and/or CMR findings suggestive of CA, a bone scintigraphy with no or mild (grade 1) cardiac uptake should be carefully interpreted together with exhaustive hematological investigations, and it should prompt clinicians to suspect AL-CA. More rarely, also patients with ATTR-CA can present with mild or no cardiac uptake at bone scintigraphy for different reasons, including early disease stage or certain transthyretin mutations like Phe64Leu and Val30Met [22, 23]. Importantly, on the other hand, it should be kept in mind that up to 20% of patients with AL-CA can show grade 2 or 3 radiotracer uptake [2, 24, 25], underlying the need for thorough hematological investigations in every patient with suspected CA. In any case, the execution of bone scintigraphy should not delay the laboratory investigations necessary to rule out the presence of a monoclonal component.

Histological demonstration of AL-type amyloid deposits is necessary in any case of suspected AL amyloidosis [1] (Fig. 2). Importantly, it should be reminded that the sensitivity of Congo Red staining for AL amyloidosis differs significantly based on specimen source, e.g., 69% sensitivity for bone marrow biopsy, 75% for fat pad aspiration, 100% for heart biopsy [26]. Thus, negative results of peripheral biopsy should not rule out AL diagnosis, especially if pre-test probability is high, as pointed out in the second case report.

Moreover, typing of amyloid deposits with mass spectrometry, immunohistochemistry, or immunoelectron microscopy remains essential [1, 5] (Fig. 3). In the third clinical case, while the patient was referred for suspected AL-CA, amyloid fibrils typing with adequate techniques revealed ATTR deposits and the patient is now being treated for this condition together with a rigorous hematological follow-up. In case of cardiac uptake at bone scintigraphy and at least one abnormal monoclonal protein test, histological confirmation with amyloid typing is recommended, usually with endomyocardial biopsy [1]. In this specific clinical case, after evidence of ATTR deposits at fat pad immunogold electron microscopy, we decided to proceed with close hematological and cardiological follow-up and to avoid for now an invasive procedure such as endomyocardial biopsy, also considering patient’s informed preference. It should be remembered, however, that cases of two concomitant types of CA, although rare, have been reported with evidence at heart biopsy of both ATTR and AL as the main amyloidogenic proteins in the sample [27, 28]. Therefore, endomyocardial biopsy should be considered and performed in selected cases [1] to achieve a definitive diagnosis.

Even though all the investigations mentioned herein are necessary for a correct diagnosis, the first essential step to diagnose AL-CA is disease suspicion. Several red flags can be helpful in suspecting the disease [1], keeping in mind that such a systemic and multiorgan disease requires a general and comprehensive approach [29]. Some red flags can be detected both in ATTR-CA and in AL-CA, whilst some clinical characteristics can be taken into consideration during the differential diagnosis amongst different amyloidosis forms [1, 5]. Patients with AL amyloidosis are usually younger than ATTRwt patients, even though this is not always the case for ATTRv [30]. Both AL- and ATTR-CA patients can present with heart failure with preserved ejection fraction, right-side heart failure, atrial arrhythmias, and “cured” systemic hypertension. Patients with AL amyloidosis usually manifest also debilitating systemic symptoms for which they might be referred to various physicians; symptoms might include weight loss, malaise, periorbital purpura and macroglossia together with renal and gastrointestinal involvement, peripheral neuropathy and/or autonomic dysfunction. Regarding electrocardiography [31], low QRS voltages are a frequent feature of CA, although more common in AL-CA [30, 32], probably due to a higher myocardial cytotoxicity of light chains. At echocardiography, LVH is usually more evident in ATTR-CA patients, occurring in AL-CA patients in more advanced clinical stages [30].

Cardiac biomarkers can be useful in the diagnostic assessment of AL-CA. Besides its role in the screening of patients with MGUS and multiple myeloma, an NT-proBNP > 332 ng/L (in the absence of renal failure and atrial fibrillation) is indicative of cardiac involvement in established AL amyloidosis when mean LV wall thickness at echocardiography is > 12 mm [13]. Recent data suggest that a combination of very low hs-cTnT (< 14 ng/L) and NT-proBNP (< 180 ng/L) can be useful in identifying patients at low risk for CA, whilst hs-cTnT > 86 ng/L could be relevant to spot those with high probability of the disease [33].

Prognostically, the severity of cardiac involvement [34], together with the depth and rapidity of hematological response to chemotherapy [35], are the main determinants of survival. Staging systems for AL-CA are available, based on cardiac biomarkers (cTn and natriuretic peptides) and sFLC values [11, 36].

Treatment of AL amyloidosis aims to reduce the production of amyloidogenic light chains by suppressing the underlying plasma cell clone; treatment regimen should be risk-adapted and depends on the degree of organ involvement, the performance status, age, and bone marrow findings [37]. Based on their risk, patients can be candidate to autologous stem cell transplant as part of the upfront therapy (with only 20% of newly diagnosed patients eligible for this treatment), or combination chemotherapy without stem cell transplant. Several effective chemotherapy regimens are nowadays available, as reported in the most recent Guidelines [37] and whose complete description is beyond the scope of this manuscript.

The role of the Cardiologists and the CA experts in the diagnostic and therapeutical process of AL-CA is certainly critical. However, patients with suspected CA might be evaluated first by other Clinicians, with the need of a widespread awareness of amyloidosis red flags [1, 5] and of the first, essential steps of the diagnostic algorithm that should be performed without delay and without uncertainties. Particularly, early diagnosis of AL amyloidosis among patients with the overmentioned systemic symptoms and/or with new onset heart failure/unexplained left ventricular hypertrophy is a game changer in the management of these individuals. A systematic and holistic approach is crucial to correctly identify and address the multiorgan impairment typical of this disease. A prompt diagnosis when the disease is at an early stage and the patient is in good clinical conditions (low-risk patients) can allow for a more aggressive and effective treatment, including autologous stem cell transplant [37]. A multidisciplinary counselling team including Hematologists and Pathologists will be then necessary for a correct interpretation of the investigations performed and to achieve the final diagnosis [10].

In conclusion, AL amyloidosis is a medical urgency that requires early diagnosis and specific treatment. Therefore, early suspicion of this condition and a correct diagnostic process is crucial for a quick referral to tertiary centers for guidelines-directed management and treatment. Some pitfalls in the diagnostic algorithm of this peculiar disease should be kept in mind and avoided to reduce diagnostic delay and improve patients’ outcome.
